# Cytochrome C Biosensor—A Model for Gas Sensing

**DOI:** 10.3390/s110605968

**Published:** 2011-06-01

**Authors:** Michael Hulko, Ingeborg Hospach, Nadejda Krasteva, Gabriele Nelles

**Affiliations:** Sony Deutschland GmbH, Materials Science Laboratory, Hedelfinger Strasse 61, 70327 Stuttgart, Germany; E-Mails: hulko@sony.de (M.H.); krasteva@sony.de (N.K.); nelles@sony.de (G.N.)

**Keywords:** cytochrome c, biosensor, thiol, prediction, model, sensing process

## Abstract

This work is about gas biosensing with a cytochrome c biosensor. Emphasis is put on the analysis of the sensing process and a mathematical model to make predictions about the biosensor response. Reliable predictions about biosensor responses can provide valuable information and facilitate biosensor development, particularly at an early development stage. The sensing process comprises several individual steps, such as phase partition equilibrium, intermediate reactions, mass-transport, and reaction kinetics, which take place in and between the gas and liquid phases. A quantitative description of each step was worked out and finally combined into a mathematical model. The applicability of the model was demonstrated for a particular example of methanethiol gas detection by a cytochrome c biosensor. The model allowed us to predict the optical readout response of the biosensor from tabulated data and data obtained in simple liquid phase experiments. The prediction was experimentally verified with a planar three-electrode electro-optical cytochrome c biosensor in contact with methanethiol gas in a gas tight spectroelectrochemical measurement cell.

## 1. Introduction

Knowledge about the chemical composition of gases can provide valuable information in fields like healthcare or environmental monitoring. The analysis of breath, for example, is an attractive topic in the field of healthcare driven by the idea of having a non-invasive diagnostic method. Sulfur compounds like methanethiol, dimethylsulphide or hydrogen sulfide in the low parts per billion (ppb) range in breath were identified as markers for bacterial infection of the oral cavity (halitosis, bad breath) [1,2]. Nitrogen oxides have been shown as marker for asthma [3]. In the field of biogas production, H_2_S concentration reflects the quality of the produced gas, thus biogas production could be controlled by using a H_2_S gas sensor. In order to get information about the chemical composition of gases many kinds of chemical sensors are under development or are already on the market [4].

Biosensors are generally developed for the detection of analytes in the liquid phase, particularly in aqueous phase that is close to the physiological environment of the biomolecules used. The strength of biosensors is their high selectivity due to the specific interaction between the biomolecule receptor and the analyte. Several examples of gas sensing biosensors exist in literature, e.g., for nitric oxide, methanethiol, or ethanol [5–7]. Using biosensors for gas phase analysis possess specific challenges mainly related to the analyte transfer from the gas to the liquid phase, and to the chemical and physical processes in which the analyte is involved. We saw the necessity to investigate the involved processes in order to facilitate the development of biosensors for gas analysis.

In this study, we divided the overall process of gas biosensing into individual steps and analyzed each step separately. Among the analyzed steps are phase partition equilibrium, chemical reactions, e.g., dissociation of the analyte after entering the liquid phase, mass-transport of analyte, and the signal generating reaction itself. A quantitative description for each step was worked out. All steps were combined into a mathematical model that allowed us to predict the biosensor response. We demonstrated exemplarily the use of the model for a cytochrome c biosensor for detecting methanethiol directly from the gas phase. In this case the prediction of the biosensor signal was derived from tabulated data of physical parameters and from experiments in liquid phase. Finally, gas phase measurements were done to demonstrate the correlation between the model predictions and the experimental biosensor response.

## 2. Material and Methods

### 2.1. Preparation of Biosensor: Cytochrome C Modified SnO_2_

For the preparation of a porous SnO_2_-layer on fluorine doped tin oxide (FTO)-coated glass (Atock Co., Ltd., Japan), the FTO slide was thoroughly cleaned by successive washing with acetone, 2% Hellmanex solution (Hellma GmbH & Co. KG, Germany), deionized water, and isopropanol. In-house prepared SnO_2_-paste was screen-printed on the FTO and sintered at 450 °C for 30 min [8]. The SnO_2_ was characterized by Scanning Electron Micrsocopy (SEM, Leo Gemini 1520) and Brunauer Emmett Teller (BET) analysis (ASAP2010, Micromeritics). The SnO_2_ particles were of 10 nm diameter. BET surface was 78 m^2^/g and pore size 16 nm (data not shown). The thickness of the SnO_2_-layer was 2.5 μm, as determined with a profilometer (KLA-Tencor, Germany). The SnO_2_-covered area on the FTO was 1 cm^2^, resulting in a geometric volume of SnO_2_ layer of 0.25 μL. The prepared FTO-SnO_2_ plates served as porous and optical transparent electrodes for addressing the cytochrome c electrochemically.

After cooling, the SnO_2_-FTO plates were immersed for 24 h at 4 °C in a solution of 2 mg/mL cytochrome c (∼12 kDa, Sigma-Aldrich GmbH, Germany) in 10 mM sodium phosphate buffer, pH 7. Like this positively charged cytochrome c was bound electrostatically to the negatively charged SnO_2_ [9,10]. All chemicals for buffer preparation were purchased from Merck (Merck KGaA, Germany).

The cytochrome c modified SnO_2_-FTO plates were rinsed thoroughly with deionized water. The sensor was coated by spin coating 35 mg/mL gelatin type B (Sigma-Aldrich GmbH, Germany) in 10 mM sodium phosphate buffer, pH 7. The thickness of the gelatin layer was measured with a profilometer to be 200 nm. The thin film of gelatin served as the electrolyte, as a protective layer for the cytochrome c, as well as an aqueous compartment in which the sensing reaction takes place. The plates were stored at 4 °C until usage.

In order to determine the overall amount of bound cytochrome c within the SnO_2_ layer it was washed off with a known volume of 3 M NaCl solution. This high salt concentration inhibited the electrostatic binding between cytochrome c and SnO_2_. The concentration of desorbed cytochrome c in a cuvette was determined photometrically by applying the Lambert-Beer law [Equation (1)]:

(1) A=ɛ⋅c⋅d

where *A* is the absorbance, *ɛ* the absorption coefficient of cytochrome c, *c* the concentration, and *d* is the optical path length. The absorption coefficient of oxidized cytochrome c at 408 nm is 1.05 × 10^5^ mM^−1^ cm^−1^ [11].

The amount of cytochrome c immobilized within the SnO_2_ layer was calculated taking into account the concentration of cytochrome c determined photometrically and the geometric volume of SnO_2_ (0.25 μL). The resulting effective bulk concentration of cytochrome c within the SnO_2_ layer was estimated to be 10 mM. We assume that the cytochrome c is homogeneously distributed in a liquid compartment with the size of the SnO_2_ layer. This is physically incorrect, but it facilitates the subsequent kinetic calculations and is an accepted and verified procedure in the field of immunological tests.

### 2.2. Reaction Rate Measurements with Immobilized Cytochrome C in Solution

The cytochrome c-modified SnO_2_ sensor plate, without gelatin, was mounted in a self-made spectroelectrochemical cell filled with 10 mM sodium phosphate buffer, pH 7. A potential of +80 mV *vs.* Ag/AgCl was applied for 20 seconds with a potentiostat (Bioanalytical System, Inc, USA) in order to oxidize the cytochrome c electrochemically. 50 μL of a stock solution of mercaptoethanol (Sigma-Aldrich GmbH, Germany) was injected such that a defined final concentration of mercaptoethanol was obtained in the buffer of the reaction vessel. Absorbance changes (ΔA) of fully oxidized and fully reduced cytochrome c at 550 nm were recorded with a Lambda35 spectrophotometer (PerkinElmer GmbH, Germany). Using Equation (1) and the concentration of 10 mM cytochrome c within the SnO_2_ (determined above), the effective absorption coefficient of reduced cytochrome c ɛ_550nm_ = 22 mM^−1^ cm^−1^ was experimentally determined. This effective absorption coefficient reflects the difference between absorption coefficient of reduced and oxidized immobilized cytochrome c at 550 nm.

### 2.3. Assembly of Electrodes on the Cytochrome C Biosensor

**Figure 1.**
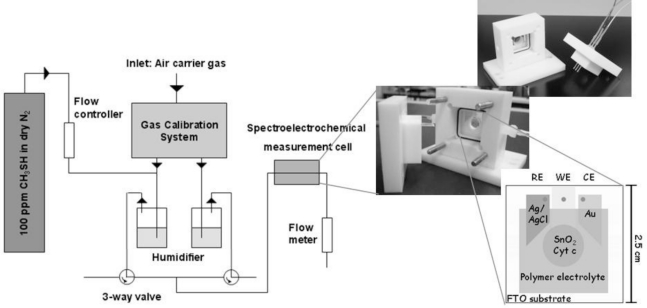
Gas-measurement set-up—A mixture of 100 ppm methanethiol in nitrogen was taken from a pressurized gas bottle. The gas was humidified and could stream into the gas-tight measurement cell. Valves allowed switching between test gas (humidified air with methanethiol) and reference gas (humidified air without methanethiol).The images show the gas-tight spectroelectrochemical measurement cell and a schematic picture of the biosensor.

For building up a planar three-electrode cytochrome c sensor, reference and counter electrodes were attached to the FTO-plate next to the cytochrome c modified SnO_2_ (working electrode). For the counter electrode gold was thermally evaporated on self-adhesive foils (CMC Klebetechnik GmbH, Germany). Ag/AgCl paste (Acheson, Netherlands) was printed on self-adhesive foil, dried at 80 °C and served as reference electrode. The electrodes were additionally coated with gelatin solution. A schematic picture of the biosensor is shown in Figure 1. The biosensor plates were stored at 4 °C until usage.

### 2.4. Gas-Phase Measurements

Planar three-electrode cytochrome c biosensor plates were mounted in a self-made gas-tight spectroelectrochemical Teflon^®^ cell that provided contact pins for electrode attachment, in- and outlets for sample gas, and an optical window for spectroscopic analysis in a photometer (see Figure 1). The inner volume of the cell was about 1 mL.

A gas mixture of 100 ppm methanethiol in nitrogen was purchased in a pressurized bottle (Linde AG, Germany). The continuous dosing with methanethiol was done using a home-made system of valves as depicted in Figure 1. Humidified air (reference) or 100 ppm humidified methanethiol in air (test gas) were provided sequentially to the measurement chamber. In order to saturate the water for humidification with the analyte, the system was equilibrated in advance for 30 min. The gas flow rate was 250 mL/min in all experiments. All measurements were performed at room temperature (∼ 22 °C).

## 3. Results and Discussion

### 3.1. Cytochrome C Biosensor

**Figure 2.**
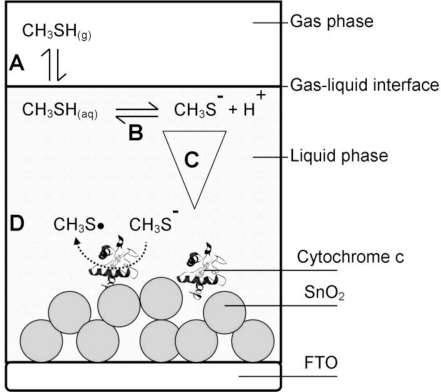
Schematic representation of the methanethiol gas biosensor—Methanethiol molecules in the gas phase are the analytes to be detected. The first step in the sensing cascade is the transfer of methanethiol from gas to liquid phase (Step **A**). Deprotonation of methanethiol occurs after being dissolved in the aqueous liquid phase (Step **B**). Initially, there is a steep concentration gradient of methanethiol across the liquid phase before methanethiol distributes evenly within the liquid phase (Step **C**). Cytochrome c that is bound to SnO_2_ on FTO reacts with methanethiolate anions, which generates the readout signal (Step **D**). The symbols in the drawing do not reflect the true scales of the represented parts of the sensor.

The basic architecture of the analyzed cytochrome c biosensor is outlined schematically in Figure 2. The gas phase sample was brought in contact with the gelatin (liquid phase) and thereby formed the gas-liquid-interface.

**Figure 3.**
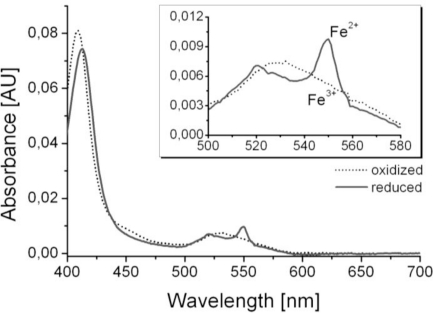
UV-spectra of cytochrome c—typical spectra of cytochrome c with three absorbance peaks of its reduced form (Fe^2+^) at 550 nm, 521 and 414 nm (solid line) and two peaks of its oxidized form (Fe^3+^) at 530 nm and 408 nm (dotted line). The inset shows an expanded view of the spectra between 500 and 580 nm.

Optical and electrochemical spectra were recorded and compared to earlier studies in order to show the functionality of the biosensing layers [12,13]. For the methodology see reference [12] and for the specific results of cytochrome c, see reference [13]. The cytochrome c modified SnO_2_-layer showed a pale orange color and UV/Vis spectra revealed the characteristic spectrum of cytochrome c comprising absorbance peaks at 408 nm and 530 nm in the oxidized Fe^3+^-state after background subtraction (Figure 3). Oxidation state of the immobilized cytochrome c was monitored by the appearance and disappearance of the typical absorbance peaks of reduced cytochrome c at 550 nm and 521 nm (Figure 3) when applying a potential sweep between −100 mV or +100 mV *vs.* Ag/AgCl. Potentials beyond this range did not result in further changes in the spectra. In cyclic voltammograms oxidation and reduction current peaks close to 0 V *vs.* Ag/AgCl were observed (data not shown) which is typical for immobilized redox proteins [12].

For thiol detection, the biosensor operation comprised two sequential steps. The first step was the electrochemical oxidation of cytochrome c to its Fe^3+^-state where the spectrum showed no peak at 550 nm. The second step was the chemical reduction of cytochrome c to its Fe^2+^-state by thiol molecules, which lead to an increased absorbance at 550 nm. The rate of increase in absorbance at 550 nm was recorded as the raw biosensor signal.

### 3.2. The Gas Sensing Process

The gas biosensor outlined in Figure 2 includes the following phases:

- **Gas phase**—contains a defined concentration of the gaseous analyte in a gas sample being in contact with the liquid phase.
- **Liquid phase**—typically consists of an aqueous liquid layer or a hydrogel covering the solid support. The biomolecule receptor can be immobilized to the solid support or can be freely dissolved. The liquid phase is in contact with the gas phase.
- **Interface**—the contact zone between gas and liquid phase.

Individual steps of the signal generating reaction processes were identified and attributed to the different phases:

- **Step A—Gas-liquid transfer:** A phase partition equilibrium of the analyte between gas phase and liquid phase.
- **Step B—Intermediate reactions:** Reactions, e.g., dissociation or hydrolysis of the analyte, which can occur after entering the liquid phase due to the new chemical environment of the analyte. These intermediate reactions can diminish the active analyte concentration or lead to products that are the actual subject of the sensing process.
- **Step C—Diffusion:** Initially there is a steep analyte concentration gradient within the liquid phase with highest concentrations at the interface and lowest concentration at the point furthest from the interface. The distribution of the analyte within the liquid phase and its diffusion rate are of crucial importance, if diffusion becomes the limiting step of the overall sensing process.
- **Step D—Signaling reaction:** Description of the actual signal generating liquid phase reaction between the biomolecule and the analyte that produces the raw signal that can be the rate of a kinetic process or an end-point at equilibrium state.

In the specific biosensor of this work the gas phase contained humidified air comprising a defined amount of gaseous methanethiol as the analyte of interest.

### 3.3. Step A—Gas-Liquid Transfer

The first step in the sensing cascade is the partition of the analyte between the gas phase and the liquid phase of the biosensor. Henry’s law [Equation (2)] allows quantification of the concentration of the analyte in the liquid phase for a given concentration in the gas phase at equilibrium:

(2) $$c=k_{H}\cdot p$$

where *c* is the concentration of the analyte in solution, *p* is the partial gas pressure, and *k_H_* is the Henry constant with the dimensions of concentration divided by pressure.

For methanethiol in an air-water system the Henry constant was found to be 0.39 M/atm [14]. We applied this value to describe the partition of methanethiol in the gelatin layer despite the fact the value was originally determined for water. Due to the high water content of the gelatin film we considered it a reasonable approximation. Thus, 100 ppm (parts per million) methanethiol at 25 °C and at atmospheric pressure in the gas sample resulted in 40 μM dissolved methanethiol in the aqueous gelatin film.

### 3.4. Step B—Intermediate Reaction

Intermediate reactions describe potential reactions of the analyte with the solvent or other dissolved compounds after being transferred from gas to liquid phase into a new and different chemical environment. Such reactions can become the rate-limiting process that requires a kinetic analysis from which the biosensor response can be derived. If these reactions are not rate-limiting then the analysis of the reaction equilibrium will be required in order to determine relevant concentrations of actual (intermediate) analyte molecules in the liquid phase.

In case of thiols it is known that their redox reactions are strongly pH dependent as the deprotonated thiolate anion takes part in electron transfer reactions [15]. The reaction between methanethiol and cytochrome c is actually a single electron transfer between the methanethiolate anion and the oxidized cytochrome c. Methanethiol itself was thus just a precursor of the actual analyte methanethiolate anion. It was therefore important to calculate the concentration of the methanethiolate anion *[CH_3_S^−^]* that is the product of the proton dissociation reaction:

$$CH_{3}SH↔CH_{3}S^{-}+H^{+}$$

The law of mass action [Equation (3)] applies:

(3) $$K_{a}=\frac{\left[H^{+}]\cdot [CH_{3}S^{-}\right]}{\left[CH_{3}SH\right]}$$

where *K_a_* is the acid dissociation constant of methanethiol (*K_a_* = 10^−10.3^ M) [16]. By assuming *[H^+^] = 10^−7^* *M*, as a buffer at neutral pH is used, *[CH_3_S^−^]* is given by Equation (4):

(4) $$[CH_{3}S^{-}]=\frac{K_{a}\cdot [CH_{3}SH]}{\left[H^{+}\right]}$$

The initial concentration of methanethiol depends on its gas-liquid equilibrium partition and was 40 μM in the liquid phase for 100 ppm in the gas phase, as shown above by using Henry’s law. Thus the methanethiolate anion concentration in the liquid phase *[CH_3_S^−^]* was 20 nM. The consequence of the intermediate reaction in this particular biosensor was significant since the sensor signal was generated by the actual analyte (methanethiolate anion) which concentration was 2000 times lower than the concentration of the compound of original interest (methanethiol).

### 3.5. Step C—Diffusion

The third step of the sensing cascade is the distribution of the analyte within the liquid phase. That requires considerations about mass-transfer rates that can become the rate-limiting step of the overall sensing process. Diffusion is the only mass-transport mechanism since convection and migration can be neglected in the given biosensor architecture. Initially, there is a steep analyte concentration gradient across the liquid phase. The concentration gradient will change over time according to Fick’s second law of diffusion [Equation (5)]:

(5) $$\frac{\partial c}{\partial t}=D\cdot \frac{\partial^{2}c}{\partial x^{2}}$$

where *c* is the concentration of the analyte in the liquid phase, *t* the time, *D* the diffusion coefficient and *x* the distance from the interface perpendicular to the interface plane. Three assumptions are generally acceptable by solving Equation (5): (i) The diffusion coefficient of the analyte in aqueous medium is independent of its concentration; (ii) Initially, before diffusion could take place the concentration of the analyte at the interface is instantly equal to its equilibrium concentration *c_0_* and zero in the rest of the liquid phase; (iii) The concentration at the interface remains constant throughout the diffusion process which is for example achieved by continuous supply of gas sample.

An appropriate solution to Equation (5) is given by Equation (6):

(6) $$c(x,t)=c_{0}-c_{0}\cdot erf\left(\frac{x}{2\sqrt{D\cdot t}}\right)$$

where *erf* is the error-function [17].

**Figure 4.**
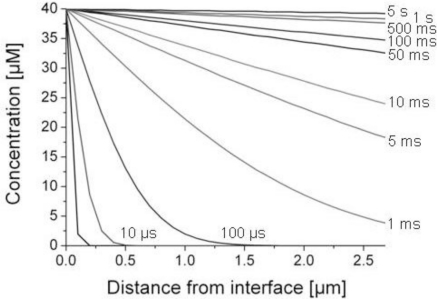
Diffusion gradients—Calculated concentration gradients of methanethiol across the liquid phase at selected time intervals after initial contact of the sample gas with the biosensor.

We applied Equation (6) to calculate how long it takes until the concentration of methanethiol in the liquid phase was above 90% of its equilibrium value *c_0_* = 40 μM. The liquid phase comprised the gelatin film of 200 nm and the SnO_2_ layer of 2.5 μm such that *x* is between 0 and 2.7 μm in our biosensor architecture. The diffusion coefficient of methanethiol was approximated to that of methanol (the alcohol analog of methanethiol) in water, D = 1.3 × 10^−9^ m^2^/s [16]. We assumed that diffusion through porous SnO_2_ is the same as for bulk liquids as shown for porous TiO_2_ layers [18,19]. This assumption is justified when no binding interactions between methanethiol and SnO_2_ occur and the pore sizes of SnO_2_ are larger than the size of methanethiol molecules. The resulting concentration profiles at selected time intervals after initial contact of the gas sample with the biosensor are shown in Figure 4. After about 500 ms the methanethiol concentration was larger than 90% of its equilibrium value anywhere in the SnO_2_ layer. By comparison of this result with the recorded sensor signal (described in the sections below) it seems that diffusion was not a rate-limiting step in the overall sensing process. If diffusion would be rate-limiting, there should be a signal plateau after a few seconds, which is experimentally not the case (see section below, Figure 6).

### 3.6. Step D—Signaling Reaction

The final step in the sensing cascade is the reaction that generates the sensor signal. In case of the cytochrome c biosensor the signal was derived from the rate of the ongoing reaction between cytochrome c and methanethiolate:

$$CH_{3}S^{-}+{\text{cytochrome}}_{\text{oxidized}}↔{CHS}^{•}+{\text{cytochrome}}_{\text{reduced}}$$

Since the reaction is bimolecular a second order reaction rate equation was an evident assumption [Equation (7)]:

(7) $$v=k\cdot \left\lfloor CH_{3}S^{-}\right\rfloor \cdot [{\text{cytochrome}}_{\text{oxidized}}]$$

where *v* is the reaction rate in μM/s and *k* is the kinetic constant in μM^−1^ s^−1^.

In order to show the correctness of Equation (7) and to calculate the kinetic constant *k* we measured reaction rates with varied starting concentrations of cytochrome c and a thiol compound. The reaction rate was photometrically measured as the formation rate of reduced cytochrome c from its oxidized form. Changes in absorbance were correlated to changes in concentration via Lambert-Beer’s law. However, a simplification of the experiment was made by applying the thiol compound dissolved in solvent instead of applying it as a gas sample. Thereby, mercaptoethanol was used instead of methanethiol because the high volatility of methanethiol prevented us from preparing solutions with accurate concentrations. We assumed that mercaptoethanol is an adequate model compound for estimating the kinetic constant of methanethiol reactions because of its close structural similarity.

**Figure 5.**
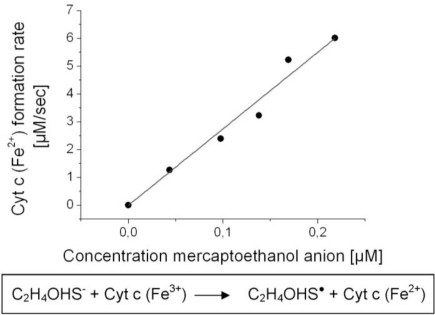
Formation rate of cytochrome c (Fe^2+^) in liquid phase plotted *vs.* mercaptoethanol anion concentrations—Cytochrome c was adsorbed onto SnO_2_ on FTO and mercaptoethanol was dissolved in buffer solution. Mercaptoethanolate reduced the oxidized cytochrome c and lead to increased absorption at 550 nm. Changes in concentration of cytochrome c were calculated from changes in light absorption via Lambert-Beer’s law.

Measured reaction rates were plotted *versus* the thiolate anion concentrations of mercaptoethanol (Figure 5). Mercaptoethanolate anion concentrations were calculated using Equation (4) from the applied mercaptoethanol concentrations and the acid dissociation constant of mercaptoethanol K_a_ = 10^−9.7^ M [16]. A linear correlation between the reaction rate and thiolate anion concentration was obtained (Figure 5), as expected by Equation (7). The kinetic constant of the redox process was calculated using Equation (7) and the concentration of cytochrome c of 10 mM: *k* = 2.8 × 10^−3^ μM^−1^ s^−1^. Analogous experiments were done in a cuvette in which both cytochrome c and mercaptoethanol were dissolved (data not shown). In this experimental setup the concentration of cytochrome c and the concentration of mercaptoethanol were varied for reaction rate measurements. The correlations between reaction rates and concentration of reactants were consistently linear, which verified Equation (7).

### 3.7. Prediction of Methanethiol Signal

Dividing the sensing process into individual steps allowed a thorough analysis of each step, as well as a prediction of the response of the cytochrome c biosensor. The prediction was made by following calculations:

- The cytochrome c concentration within the SnO_2_ layer was 10 mM, as given by the sensor preparation procedure;
- The concentration of methanethiol in the liquid phase was 40 μM at a gas phase methanethiol concentration of 100 ppm, as determined by applying Henry’s law [Equation (2)];
- Methanethiol distributes by diffusion within the liquid phase and would reach 90% of its equilibrium value within 500 ms [Equation (6)];
- Deprotonation of methanethiol to methanethiolate occurs as an intermediate reaction, the concentration of the actual analyte methanethiolate anion would be 20 nM [Equation (4)]. A homogeneous distribution of methanethiolate within the liquid medium was assumed;
- The reaction rate of cytochrome c reduction by methanethiolate would be *v* = 0.6 μM/s, calculated by using Equation (7) and the estimated kinetic constant *k* = 2.8 × 10^−3^ μM^−1^ s^−1^ (see Step D—Signaling reaction);
- Lambert-Beer’s law allowed converting the reaction rates to absorbance change rates of the biosensor of 2.0 × 10^−4^ in absorbance units per minute. Absorbance change per minute = *v* * Δɛ * d * 60 = 0.6 µM/s * 22 mM^−1^ cm^−1^ * 2.5 μm * 60 s. This would be the expected sensor signal that has been subjected to experimental verification.

### 3.8. Experimental Verification

**Figure 6.**
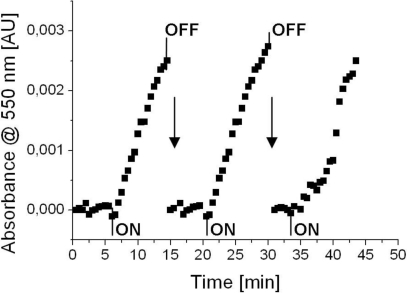
Gas biosensor measurements—with a cytochrome c modified electrode and gaseous methanethiol. The changes in absorbance at 550 nm are plotted *versus* the time of the experiment. The moments when reference gas was switched to methanethiol-containing gas sample are marked with “ON”, whereas “OFF” indicates switching from methanethiol-containing gas sample to reference gas. The vertical arrow indicates electrochemical oxidation of cytochrome c (60 s, +100 mV *vs.* Ag/AgCl) to reset to baseline absorbance and to prepare the biosensor for the next exposure of gas sample.

Finally, the predicted reaction rate had to be verified experimentally. Therefore, gas measurements were conducted with the planar cytochrome c biosensor. The absorbance of cytochrome c at 550 nm was recorded *versus* time for three subsequent exposures of methanethiol (Figure 6). At the beginning cytochrome c was oxidized electrochemically (60 s., +100 mV *vs.* Ag/AgCl) to its Fe^3+^-state. A stable baseline could be observed when reference gas flowed through the chamber. Immediately after switching to test gas with methanethiol, an increase in absorbance at 550 nm could be observed. This was caused by methanethiol reducing cytochrome c to its Fe^2+^-state. The points in Figure 6, where absorbance dropped sharply, were caused by electrochemical oxidation of cytochrome c (60 s., +100 mV *vs.* Ag/AgCl) in order to prepare the biosensor for the next exposure of methanethiol and to reset the baseline level to zero. The averaged absorbance changes during methanethiol exposure were 3.6 × 10^−4^ ± 0.4 × 10^−4^ absorbance units per minute. Comparing this value with the predicted value of the model (2.0 × 10^−4^ absorbance units per minute) we can conclude that the value is in the same order of magnitude and was therefore regarded as experimental verification of the predicted value. However, we have to state that we did not consider the error of the model itself. Different inaccuracies, for example by taking model compounds or other assumptions made during defining the model, may add to an error of such a model.

## 4. Conclusions

In the field of gas biosensor development the gas-liquid interface between the gas sample and the aqueous part of the biosensor is unique and poses special challenges. In order to obtain better understanding of the sensing process we divided the process into individual steps and analyzed them individually. Finally, we combined them into a mathematical model for making biosensor response predictions. Our conclusion is based on the experimentally verified response prediction of a cytochrome c biosensor for sensing methanethiol in a gas sample. This information allows focusing on crucial issues and will facilitate biosensor development.

The general nature of the suggested model allows its broad applicability to a great variety of biosensor architectures. We showed how the abstract model can be adapted to a particular biosensor, the cytochrome c biosensor for methanethiol detection in gas samples.

Besides the analysis of the sensing process, we demonstrated to our knowledge for the first time the detection of thiols directly from a gas sample with a cytochrome c biosensor. Cytochrome c modified SnO_2_ on FTO covered with a protecting film of gelatin proved to be a suitable sensor platform. SnO_2_ on FTO as transparent electrode allowed probing the redox state of the immobilized cytochrome c by optical spectroscopy. Additionally cytochrome c could be brought electrochemically to a defined redox state at any time during the process. Cytochrome c proved to be robust and withstood all preparation and process steps. However, the pH of the aqueous sensor compartment had to be kept around pH 7 and salt concentrations had to be kept at physiologic concentrations or below to prevent desorption of cytochrome c from the surface. These restrictions prevented sensor measurements at higher pH values for which higher reaction rates could be expected due to the pH dependency of methanethiol deprotonation. The gelatin layer was successfully applied as water reservoir that served as both: Aqueous environment for methanethiol and cytochrome c, and electrolyte to connect the electrodes in a planar three-electrode sensor layout. Furthermore, we found that the quality of such a cytochrome c biosensor is good enough for repetitive measurements during 60 min. Even the regeneration of a dried out sensor is possible by supplying a humid environment. Generally, reducing or oxidizing compounds in the gas sample could interfere with the methanethiol measurement, if reacting with cytochrome c. However, even a low selectivity of cytochrome c has been observed, oxygen for example did not interfere with the reaction at all. These aspects are important for the applicability in industrial settings and would be part of sensor validation during a development phase.
